# Aptamer Applications in Emerging Viral Diseases

**DOI:** 10.3390/ph14070622

**Published:** 2021-06-28

**Authors:** Arne Krüger, Ana Paula de Jesus Santos, Vanessa de Sá, Henning Ulrich, Carsten Wrenger

**Affiliations:** 1 Department of Parasitology, Institute of Biomedical Sciences, University of São Paulo, São Paulo 05508-000-SP, Brazil; krueger.arne@icb.usp.br; 2 Department of Biochemistry, Institute of Chemistry, University of São Paulo, São Paulo 05508-900-SP, Brazil; ana_paulajs@hotmail.com (A.P.d.J.S.); vankaren@gmail.com (V.d.S.)

**Keywords:** aptamers, SELEX, SARS-CoV-2, viral infections, aptasensor, aptazyme

## Abstract

Aptamers are single-stranded DNA or RNA molecules which are submitted to a process denominated SELEX. SELEX uses reiterative screening of a random oligonucleotide library to identify high-affinity binders to a chosen target, which may be a peptide, protein, or entire cells or viral particles. Aptamers can rival antibodies in target recognition, and benefit from their non-proteic nature, ease of modification, increased stability, and pharmacokinetic properties. This turns them into ideal candidates for diagnostic as well as therapeutic applications. Here, we review the recent accomplishments in the development of aptamers targeting emerging viral diseases, with emphasis on recent findings of aptamers binding to coronaviruses. We focus on aptamer development for diagnosis, including biosensors, in addition to aptamer modifications for stabilization in body fluids and tissue penetration. Such aptamers are aimed at in vivo diagnosis and treatment, such as quantification of viral load and blocking host cell invasion, virus assembly, or replication, respectively. Although there are currently no in vivo applications of aptamers in combating viral diseases, such strategies are promising for therapy development in the future.

## 1. Introduction

The human population is ever-growing, which correlates with an increasing quantity of related diseases. Augmenting pathogen biomass and interaction with several hosts raise the chances of mutation and thus drug resistance. As such, novel diagnostic and therapeutic tools to tackle the foreseeable problems of the next generation are urgently needed [1,2,3].

Infectious diseases are associated with public health problems usually caused by pathogens, such as the Severe Acute Respiratory Syndrome virus (SARS-CoV), Middle East Respiratory Syndrome virus (MERS-CoV), SARS-CoV-2, and influenza viruses. The early detection process is a requested solution to diagnose, treat, and eliminate the uncontrolled spread of the virus in each population [4]. As viruses undergo genetic modification, previous global outbreaks showed a rapid surge of deaths worldwide in 1918 because of H1N1 virus and 1957 by H2N2 virus, in 2003 due to SARS-CoV, in 2009 by H1N1 pdm09 virus, in 2012 by MERS-CoV, in 2014 by Ebola virus, and since 2019 by SARS-CoV-2 infections [5,6]. The presence of the virus must be detected by biochemical analysis to prevent the spread of infection and ultimately reduce the death toll. Thus, it is necessary to develop diagnostic methodologies that are quick and simple, highly effective, specific, and sensitive. Currently, the identification of viral particles and nucleic acid is generally performed by enzyme-linked immunosorbent assay (ELISA) and polymerase chain reaction (PCR) methods, respectively [7].

Pathogen detection is the fastest growing area involving aptamers to identify a specific target using viral molecular biomarkers [8]. However, not only is the development of new tools important, but also—and becoming even more prominent—the commercial value in terms of their development, maintenance, and application costs.

**Figure 1:**
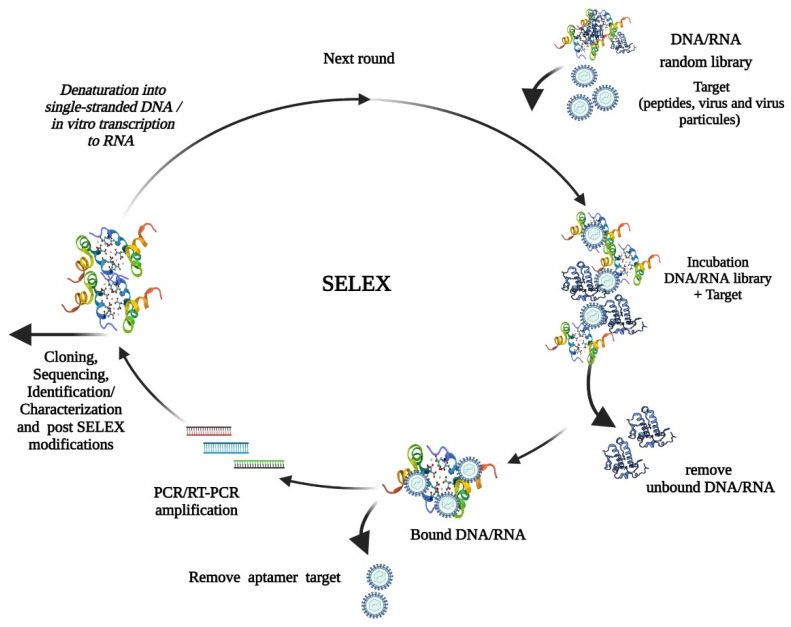
Schematic representation of in vitro SELEX. The combinatorial DNA or RNA library is incubated with the target complex (e.g., magnetic beads coupled with streptavidin + biotin-labeled virus pool) for ligation. The procedure is characterized by the repetition of the successive steps consisting of selection (binding, washing, and elution), amplification, and purification. In the first round, the library and the target molecules are incubated for binding. Unbound oligonucleotides are removed, and the bound target oligonucleotides are eluted and subsequently reverse transcribed by RT-PCR (RNA), following the amplification by PCR (DNA). A new enriched pool of selected oligonucleotides is generated by a DNA preparation for in vitro transcription. The selected pool is used for the next round of selection. In the last round, the pool of enriched aptamers is cloned and sequenced, and afterwards, several individual aptamers are characterized. Created with BioRender.com (License #2364-1511, Toronto, ON, Canada).

Aptamers are DNA/RNA oligonucleotides selected in a repetitive in vitro screening procedure called Systematic Evolution of Ligands by EXponential Enrichment (SELEX). They are obtained from consecutive SELEX cycles that use a chemically synthesized initial random library, consisting of 10^12^–10^15^ short, single-stranded RNA or DNA sequences. Aptamer selection (Figure 1) occurs by binding DNA/RNA oligonucleotides to a biomolecule target until they are narrowed down to a nearly homogeneous population of high-affinity target binders [9,10]. The identification and characterization of high-affinity aptamers are carried out by DNA sequencing, in which the identified ligands are clustered into groups by sequence and structure similarities and tested for their binding and biological activities to the target [11,12]. There are a variety of aptamers for different targets, selection procedures, analytical, diagnostic, and therapeutic approaches due to their advantages over antibodies. These include aptamer stability, affinity, low immunogenicity, low cost, resistance to temperature, and potential chemical modifications [13,14].

Due to their 2′-OH group, unmodified RNA aptamers are much less stable and are degraded within minutes in biological fluids, such as human serum, when compared to unmodified DNA aptamers, which may resist enzymatic degradation to 30–60 min [15]. However, inclusion of 2′-OH group pyrimidine nucleotides, in which the 2′-OH group was substituted by 2′-F or 2′-NH_2_ groups for in vitro transcription reactions, produces nuclease-resistant aptamer transcripts [16,17]. Single-stranded RNA molecules are naturally produced and are known for easily creating stem loops and tertiary structures. For instance, microRNAs tightly interact with their binding partners for post-transcriptional gene expression regulation and crucial cellular functions [18]. In view of that, it is plausible that RNA aptamers more easily produce unique tertiary structures, compared to DNA aptamers, since DNA naturally forms double-stranded structures. RNA aptamers may form more compact and a greater variety of tertiary structures due to non-Watson-Crick base pairing [19], with high binding affinity and suitability for therapy, and they might more easily enter cells, when compared to DNA aptamers [20]. However, DNA aptamers have certain advantages over RNA aptamers, such as easier and less cost-intensive production, due to the omission of reverse transcription and in vitro transcription steps [21]. A further major advantage of DNA aptamers is the easiness of introducing post-SELEX modifications by PCR reactions in the presence of primers, which carry on their 5′- or 3′-termini fluorescent probes, nanoparticles, or further moieties for aptamer applications in diagnostic or in vivo applications [22]. Although in recent years mostly DNA aptamers were developed, as yet, it depends on the respective application to decide if a DNA or RNA aptamer would be appropriate. Below, we will discuss some aptamer modifications for optimizing their use in virus diagnosis and anti-virus applications.

**Figure 2:**
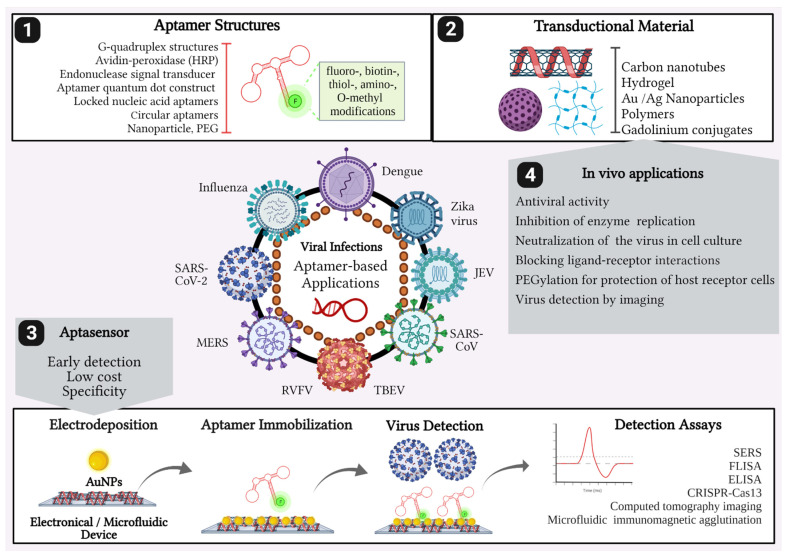
Aptamer applications for emerging viral infections. Aptamers selected by in vitro SELEX methodology used for diagnosis of several human pathologic viruses including Influenza, MERS, SARS-CoV, SARS-CoV-2, Dengue, Zika virus, Rift Valley fever virus (RVFV), Japanese Encephalitis virus (JEV), and Tick-borne encephalitis virus (TBEV). (**1**) Aptamers with modified structures can be immobilized using (**2**) engineered transduction material, such as, i.e., Au-nanoparticles (AuNPs) based on devices for detection assays, as (**3**) aptasensors, or used for (**4**) in vivo studies for inhibiting cell infection or virus replication. PEG = Polyethylene glycol. Created with BioRender.com (License #2364-1511, Toronto, ON, Canada).

The SELEX technique has already been used for the selection of aptamers aiming to combat cancer, autoimmunological, and neurodegenerative as well as infectious diseases, such as bacterial and protozoal infections, among other applications [22,23,24,25,26,27,28,29]. However, there is another, more neglected group of therapeutic targets that are related to viral infections. So far, no aptamer treatment of viral diseases has passed clinical trials, although aptamers were reported in the detection of virus particles or even their functional inhibition [30,31]. However, a sensitive and simple innovative tool called aptamer-based sensor (aptasensor) has been used for diagnostic purposes to detect infectious diseases in clinical applications (Figure 2) [32]. These biosensors are electrochemically designed to transduce signals to detect microorganisms and viral proteins through their affinity for aptamer molecules [33]. Therefore, aptamers can be employed to solve detection problems, i.e., late detection and non-distinction between infected and non-infected cells, but also to treat diverse infections [31].

Aptasensors and other devices are examples to overcome the (time-consuming) efficiency and cost limitations of conventional methods for detecting virus infection, which include PCR, reverse transcription-PCR, immunoblotting, and immunofluorescence assays [34]. Several publications have already reviewed the fundamental mechanisms of aptamer techniques, as well as applications in diagnosis and therapeutics of viral diseases [4,31,35,36,37,38]. Therefore, we will discuss the latest developments of aptamer applications in emerging viral diseases with a special focus on diseases related to coronaviruses.

## 2. Aptamers Targeting Influenza Virus

Influenza viruses are enveloped and belong to the *Orthomyxoviridae* family. The viral subtypes A, B, and C, and the immunogenic proteins of the influenza virus are defined by the surface glycoproteins haemagglutinin and neuraminidase. The genome of A and B type influenza is characterized by a segmented, negative-strand RNA consisting of 8 segments which encode for 11 proteins; in contrast, influenza C viruses hold only 7 segments. Influenza viruses A can infect mammals and are responsible for most symptomatic infections in humans, whereas influenza viruses B infect solely humans and type C influenza viruses infect pigs [39,40,41]. Influenza viruses cause three to five million severe infections each year, leading to roughly 600 thousand deaths [42].

The challenge for diagnosis of influenza viruses is related to the diversity of existing glycoproteins—usually hemagglutinin antigen—present on the surface of the different influenza subtypes A, B, and C. High rates of mutations produce more than 18 types of HA, making this information essential for the diagnosis of different strains of influenza and their effective treatments [43,44]. Seasonal and pandemic respiratory infections are frequently caused by influenza viruses. The virus subtypes A (H1N1 and H3N2 strains) and B (Victoria and Yamagata lineages) only affect humans, however, the strains of avian influenza virus (H5N1 and H7N9) have also infected and killed people during recent epidemics [45]. Therefore, developed aptamers have been used to facilitate the diagnosis of Influenza A viruses since 2009, when Tam et al. created a DNA aptamer sensor with carbon nanotube binders (MWCNTs). These nanotube binders were constructed to be adhered to electrode devices by electrostatic forces and they demonstrated the immobilization of an aptamer–nanotube complex by Raman spectra analysis. Aptamer detection was based on double-stranded DNA interaction: a link between the DNA aptamer immobilized on the sensor and the presence of a DNA influenza A sample showed a detection limit of 0.5 nM [46].

More recently, the detection of influenza A strains has been described using an aptasensor based on surface-enhanced Raman spectroscopy (SERS). The group developed a device to improve the quality of the influenza A aptamer technique by introducing SERS for signal amplification. Quantitative diagnosis for influenza A viruses was achieved by using colloidal silver nanoparticles (AgNP). Two types of DNA aptamers were incubated in aqueous AgNP environment (used as the base of the sensor): aptamers RHA0385 and BV42, with an affinity for a variety of HA antigens of influenza A strains. These aptasensors detected influenza A virus particles and showed an innovation of quantitative detection for SERS-based assays, proving that DNA–RNA interactions (aptamer and viral particles, respectively) are more effective for this diagnostic analysis when compared to DNA–DNA interactions [47].

**Figure 3:**
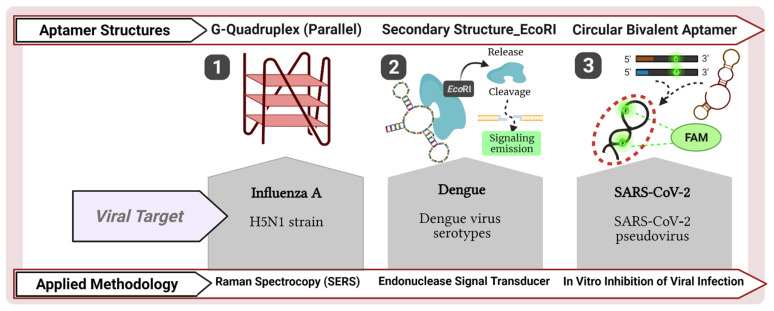
Aptamer structures for the detection of emerging viral diseases. (**1**) Four-stranded DNA structure of RHA0385 aptamer developed to interact with HA antigen of influenza A strains (this aptamer was first selected for binding to the H5N1 influenza virus). Conformational changes of the quaternary structure are analyzed by surface-enhanced Raman spectroscopy (SESRS) detection, which amplifies the signal of aptamer–RNA virus interaction in colloidal silver nanoparticles (AgNP). (**2**) Secondary structure of an aptamer with affinity for Dengue virus serotypes. A biosensor is used to detect the presence of the target virus using aptamer coupled to endonuclease EcoRI. After the recognition of Dengue virus serotypes, EcoRI is released from the aptamer structure and will act as an endonuclease signal transducer to cleave a signaling molecule, turning it fluorescent for the detection assay. (**3**) Circular bivalent aptamer of a predicted secondary structure of an inhibitory aptamer specific to SARS-CoV-2 pseudo-virus. Two components, which are both carboxyfluorescein (FAM)-labeled, of CoV2-6C3 aptamer are dissolved in a T4 DNA ligase solution under different temperature conditions to construct the circular bivalent aptamers. The circular aptamer structures prevent degradation by exonuclease activity, and its two recognition motifs promote blocking of SARS-CoV-2 infection in pseudo-virus neutralization assay, as verified by fluorescence emission. All images were adapted from [48,50,51] and created with BioRender.com (License #2364-1511, Toronto, ON, Canada).

Following the generic detection of influenza A viruses, the RHA0385 aptamer designed to recognize the viral HA antigen was studied according to its tertiary aptamer structure related to conformational changes when interacting with the target protein of different strains. The RHA0385 aptamer and its derivatives maintain the specific G-quadruplex structure for the HA sites of influenza A viruses (Figure 3(1)). Therefore, conformational changes in the RHA0385 G-quadruplex-aptamers can be explored to diagnose influenza A viruses based on different varieties of HA antigens [48]. In 2015, an aptasensor was discovered to detect the HA gene using tetrahedral nanostructured probes. The group worked with DNA aptamers composed of the assembly of three thiolated nucleotides and one oligonucleotide strand with a sequence complementary to its H7N9 strain targeting the avian influenza virus (AIV). The tetrahedral aptamer was hybridized to a gold electrode surface and improved signal detection (detection limit of 100 fM) by conjugating avidin-horseradish peroxidase (HRP) to the aptamer structure [49].

Aptasensors have been used as an electrochemical diagnostic tool with advantages such as early detection, low cost, and specificity for influenza A and HA glycoprotein viral particles. Therefore, aptamers have been identified to bind with affinity to HA antigens from different strains of influenza. For further applications in diagnosis and possibly therapy, aptamers can be modified with biotin [52,53], amine groups [54], fluorophores [55,56,57,58], and thiol groups that will be attached to nanostructured materials, such as polymers [59,60], gold nanoparticles [61,62,63], metallic semiconductors [64,65], carbon-nanotubes [66,67], nanocomposites [68], and microspheres [69].

## 3. Aptamers against Dengue

Arboviruses (Arthropod-borne viruses) are RNA viruses (except for the African swine fever virus, the only known DNA arbovirus) and pose an important global health problem. Arboviruses are characterized by a broad variety of RNA viruses, which include the alphaviruses, the flaviviruses, the bunyaviruses, the orbiviruses, the vesiculoviruses, the nairoviruses, the phleboviruses, and the thogotoviruses [70,71].

Flavivirus infections are a significant public health problem, since several members of the *Flaviviridae* family are highly pathogenic to humans, such as the Dengue virus (DENV), the Zika virus (ZIKV), Yellow Fever virus (YFV), Tick-borne encephalitis virus (TBEV), and West Nile virus (WNV), among others. Flaviviruses share some common characteristics, they are of a defined size (40–65 nm), symmetry (enveloped, icosahedral, nucleocapsid), and the genome is a single RNA strand with positive polarity of about 10,000–11,000 bases. In general, the genome encodes three structural proteins (Capsid, prM, and Envelope) and seven non-structural proteins (NS1, NS2A, NS2B, NS3, NS4A, NS4B, and NS5), which are important for regulating and expressing the virus, such as replication, virulence, and pathogenicity [72,73,74].

As outlined above, the DENV also belongs to the *Flaviviridae* and is represented by the four serotypes, DENV-1 to DENV-4. DENV infections can cause serious symptoms such as myalgia, headache, retro-orbital pain, and hemorrhagic fever [75,76].

In recent studies, thioaptamers have been generated to target a recombinantly expressed area of the Dengue virus type-2 envelope protein [77]. After applying SELEX, filter-binding assays demonstrated that the selection of the thioaptamers was successful, with K_D_ values from 99 to 180 nM for target protein binding. Furthermore, the authors could show that some of their aptamers neutralized the virus, which could lead in the future to the design of novel potent inhibitors.

In a different study, DNA aptamers against the four serotypes of DENV have been selected for a potential application as antivirals [78]. In particular, the DENV E protein is a promising target which consists of three different domains, EDI, EDII, and EDIII, of which the latter has been suggested to be involved in the attachment of the virus to the receptors of the respective host cell membrane. By genetic alteration of the EDIII coding region, subsequently, mutated recombinantly expressed proteins have been obtained, which were subject to aptamer selection. Chen and colleagues identified an aptamer, S15, that performed very well in a plaque reduction neutralization test assay and had antiviral activity against all four serotypes of DENV [78].

Aptamer applications are not only restricted to interference with virus proliferation, which is a fundamental factor, but are also important for the discovery of novel rapid diagnostic methods to identify and quantify the amount of DENV.

An interesting diagnostic test has been developed by Fletcher et al., who developed a modular biosensor that can rapidly identify sequences of the DENV genome. This biosensor consists of an oligonucleotide linker module, an aptamer/EcoR1 restriction endonuclease signal transducer, as well as a fluorescent signaling molecule (Figure 3(2)). In several consecutive steps, the restriction endonuclease is released from the aptamer and rapidly cleaves several signaling molecules to obtain a detectable signal. With this technique, the authors were able to develop a robust dengue serotype-specific biosensor without any cross-reactivity that is easy to use and of potentially low reagent costs [50].

## 4. Aptamers against Rift Valley Fever Virus

In sub-Saharan Africa, the Rift Valley fever virus (RVFV) is endemic, a mosquito-borne bunyavirus belonging to the genus *Phlebovirus*. The virus has even spread to Egypt [79], and due to the presence of appropriate mosquito vectors in Europe, Asia, and the Americas, the virus might have the potential for a global spread. Currently, no specific treatment for RVFV infection is available and one of the few treatments of viral hemorrhagic fevers is the use of Ribavirin, a nucleoside analogue, however, the tremendous side effects limit its application [80,81,82].

RVFV holds a tripartite, single-stranded, negative-sense RNA genome that encodes seven proteins. The RVFV nucleocapsid protein N is an RNA-binding protein required for the assembly of viable viruses and is involved in several stages of viral replication. During replication, the RVFV N protein encapsulates viral genomic and antigenomic RNA [83]. RNA aptamers have been generated that bind to the respective N protein with high affinity. Additionally, the group of Lodmell was able to label an RNA aptamer with fluorescein. Competitive binding experiments showed the aptamer specificity and demonstrated their potential in the generation of a sensitive fluorescence-based sensor that can be used in further diagnostic or drug-screening applications [83].

## 5. Aptamers against the ZIKA Virus

A few years ago, ZIKA virus infection became a serious health problem in Brazil. In this sense, specific diagnostic tools have been developed to detect the respective virus infection. Additionally, the aptamer technique has been employed using an aptamer-based ELISA for the highly specific and sensitive detection of Zika NS1 protein. After applying SELEX on the NS1 protein, two aptamers have been identified which were further analyzed by bioinformatics tools, and subsequently, two truncated versions have been generated. Whereas one aptamer set showed cross-reactivity against non-ZIKV NS1 proteins, the other aptamer set did not reveal any binding affinity for any of the four serotypes of DENV NS1 proteins, demonstrating its high specificity against the ZIKV NS1 protein. None of the two aptamers showed any cross-reactivity against non-related proteins, i.e., serum proteins. In the future, the specific aptamer might be useful in diagnostic applications [84].

An interesting work is based on the development of a diagnostic assay utilizing aptamer peptides to detect the ZIKV in serum and urine, which do not consist of nucleic acids such as DNA or RNA [85]. These are small peptide sequences that are designed by bioinformatics programs. In this study, a total of 25 peptide aptamers were selected, their ability to bind to ZIKV epitopes was evaluated in a molecular docking study, and one aptamer peptide was selected and further modified. In order to verify the aptamer peptides, a monoclonal antibody against the ZIKV envelope protein was generated, and both were fused to europium nanoparticle beads and analyzed in a fluorescence-linked sandwich immunosorbent assay (FLISA). The aptamer peptides as well as the antibody were able to specifically detect the Zika protein, whereas the respective controls, the DENV and chikungunya virus (CHIKV), were not detected.

A serious problem in diagnostics is the rapid differentiation between both infections, ZIKV and CHIKV. ZIKV infections are usually asymptomatic, although sometimes rash, fever, headache, vomiting, and joint pains are observed [86,87]. These symptoms overlap significantly with CHIKV infections. Additionally, both viruses can be transmitted by the same mosquito vector, therefore an early adequate diagnosis of ZIKV poses a serious challenge, especially where both the viral infections are endemic [88].

The work by Saraf et al. describes the development of a polydimethylsiloxane-based microfluidic device for the multiplex detection of ZIKV and CHIKV envelope proteins on a single platform using aptamer–analyte interactions. The current sensor is designed on a sandwich morphology of aptamer 1–antigen–aptamer 2 for the detection of ZIKV and CHIKV antigens. In this construction, aptamer 1 is immobilized on the microfluidic channel for virus protein captures, whereas aptamer 2 is conjugated to gold nanoparticles (Apt-AuNPs). By applying a silver staining technique, the AuNPs serve as electron-transferring agents in the proximity of silver ions. This process leads to continuous deposition of silver over gold, which produces a grey color in the testing zone that depends on the concentration of analyte-bound Apt-AuNPs over the channel surface. The authors narrowed the sensitivity down to clinically relevant concentrations of ZIKV and CHIKV envelope proteins in phosphine-buffered saline (1 pM) and calf blood (100 pM) [89].

The present technique provides a quantitative and qualitative strategy for detecting virus antigens. The major advantage of using an antigen-capture sandwich assay is high sensitivity and colorimetric detection capabilities with crude sample preparation [90]. The approach is similar to the ELISA technique but avoids the use of expensive plates and plate readers and is very easy to handle. Additionally, the replacement of antibodies with aptamers makes this technique more robust while maintaining the same level of accuracy and reliability of the approach.

## 6. Aptamers against Japanese Encephalitis Virus

Japanese Encephalitis virus (JEV) belongs to the *Flaviviridae* family and reveals the structural and genomic properties, as already described above. Infection with JEV confers lifelong immunity, which has also been exploited by the generation of potent vaccines. Currently, there are three vaccines based on the genotype III virus available: SA14-14-2, IXIARO, and ChimeriVax-JE [91]. Focus has also been placed on anti-JEV therapy using aptamers capable of blocking the MTase enzyme. MTase is involved in cap methylation of flavivirus RNA, which is an essential process for viral replication. JEV replicates in the cytoplasm and uses its own MTase and S-adenosyl–L-methionine as a methyl-donor for RNA capping by transferring respective methyl groups to both the N-7 position on the cap and the 20-OH position of ribose on the first transcribed nucleotide. Any interference with the RNA capping process might lead to an inhibition of virus proliferation. The group of Han and Lee [92] selected aptamers against the respective recombinantly expressed virus protein and a human methyltransferase (hRNMT) as a selective control. After 15 rounds of selection, an aptamer has been identified which interfered with the enzyme activity of the MTase by blocking methylation of the N-7 as well as the 20-OH groups. Further, the authors showed that the aptamer could inhibit the production of JEV in the host cell. Additionally, this aptamer was shown to selectively bind and thereby inhibit the JEV MTase, but not the human hRNMT counterpart, which makes this aptamer an interesting potential antiviral agent for the treatment of JEV. Since other flaviviruses encode for a similar enzyme, this approach might be useful to also target DENV, WNV, YFV, and ZIKV.

In subsequent studies, the groups of Han and Lee and Jung created RNA aptamers also against the DENV MTase. They identified two aptamers that showed almost complete inhibition of methylation, and after further optimization, a single aptamer remained which was potent against all DENV serotypes and might be useful as a future antiviral against DENV [93].

## 7. Aptamers against Tick-Borne Encephalitis Virus

Another member of the flaviviruses is the Tick-borne encephalitis virus (TBEV), which targets the central nervous system [94]. TBEV has also been subject to a SELEX-based study. In this work, aptamers were selected against the *Escherichia coli* recombinantly expressed E protein of TBEV [95]. The obtained aptamers were subsequently tested against an isolate of the Siberian TBEV in pig embryo kidney cell culture for neutralization analysis. The authors found a similar neutralization index employing their aptamers as by virus neutralization using commercial human immunoglobulin against TBEV, which is currently frequently used in TBEV diagnostics.

## 8. Aptamer Applications in Coronavirus Diseases

In 2019, a novel coronavirus emerged in China as a human pathogen and spread over the globe within weeks. Severe acute respiratory syndrome coronavirus 2 (SARS-CoV-2) causes the coronavirus disease 2019 (COVID-19), which developed into the current pandemic with a devastating impact on public health [96]. Together with the SARS-CoV and Middle East Respiratory Syndrome coronavirus (MERS-CoV) epidemics, this raised awareness of coronaviruses formerly only known to cause severe disease in animals or as the causative agent of the common cold [96,97].

Coronaviruses (CoV) comprise a large number of viruses that infect animals and humans and, therefore, are of socio-economic importance and a public health concern [98,99]. CoVs belong to the order Nidovirales and were first taxonomically divided based on serology. Applying genetic methods, CoVs were later allocated to the family of *Coronaviridae* and the subfamily *Orthocoronavirinae,* which is further categorized into groups [100,101,102]. While *Alphacoronaviruses* and *Betacoronaviruses* infect mammals, *Gammacoronaviruses* and *Deltacoronaviruses* primarily infect birds [103]. CoVs are classified as single-stranded, positive-sense RNA viruses, with the largest genome among RNA viruses [104,105]. Human CoVs were first isolated by a group of virologists in 1965 from patients with the common cold [106]. Later, the same group observed the virions under the electron microscope and termed them “corona” (Latin crown) due to the virus’ characteristic visual appearance resembling the solar corona [107]. Four distinct structural proteins are essential for virion function and architecture. Nucleocapsid proteins form a flexible, helical structure containing the RNA genome, whereas the membrane contains the envelope protein (E), the transmembrane protein (M), and the petal-shaped glycosylated spike protein (S) responsible for the virus’ name [101].

Coronaviruses are subject to a high rate of genetic variation, including a high frequency of RNA recombination and mutation resulting in the emergence of different strains and even genotypes [104,108]. This adaptability enables them to cross species borders and undergo genetic recombination with other virus species. In fact, the original hosts of CoVs are suspected to be either bats or birds, presenting the highest diversity of virus species [103]. While guaranteeing survival of the virus, this poses a major challenge for diagnostics, treatments, and even vaccination efforts, as well as a threat to public health [108]. In total, seven human CoVs are known to cause diseases of varying severity. HCoV-229E and HCoV-OC43 are responsible for mild respiratory diseases, such as the common cold, distributed globally and known since the mid-1960s [99,100,106]. When severe acute respiratory syndrome coronavirus (SARS-CoV) started to spread in China in 2002, CoVs gained attention due to the severity of the disease, with a mortality rate of ~9% [97,99,105]. As a response, research for human pathogenic coronaviruses was intensified and resulted in the detection of two additional species, HCoV-HKU1 and HCoV-NL63 [97,100,101]. In 2012, Middle East Respiratory Syndrome coronavirus (MERS-CoV) emerged in Saudi Arabia exhibiting an even more severe cause of disease, resulting in a mortality rate as high as 36% [99]. After SARS and MERS, COVID-19 emerged at the end of 2019 in China as the third human pathogenic coronavirus disease and caused a global state of emergency [109]. Since its beginning, numerous research articles have shed light on the different aspects of the pandemic, including mechanisms of SARS-CoV-2 pathogenesis. Much like SARS-CoV, human angiotensin-converting enzyme 2 (ACE2) is the receptor for the SARS-CoV-2 viral spike protein, mediating virus cell entry [96]. The main methods for the detection of the virus are either RT-PCR or serological tests. While both are established methods that work well, they present specific disadvantages. RT-PCR tests can detect the smallest amounts of virus RNA in the acute phases, but fail to distinguish between infectious or non-infectious virus. In addition, sample preparation is not trivial and requires advanced infrastructure and resources [110,111]. Serological tests on the other hand, such as ELISAs, can only detect antibodies that are present in the later stages of the infection. Additionally, the concern of false positive results due to cross-reactivity with other coronaviruses was raised [97,110]. Direct testing of virus proteins such as N and S by monoclonal antibodies are expensive, although effective [110]. Recently, biosensors have gained importance in diagnostics due to their advantageous properties. In this context, especially aptamers became promising biomolecules as novel theranostics due to their aforementioned superior properties [4,112].

Many publications have focused either on the detection of SARS-CoV-2 by diverse methods (for an extensive list, see [5], or specifically reviewed aptamers-based applications in viral disease diagnostic and therapeutic intervention [110,112,113]). Therefore, the focus here lies on the recent developments, specifically for aptamers-based technologies for SARS-CoV-2 diagnostics and treatment, reviewing some milestone aptamers applications for SARS-CoV (Table 1).

Ahn and colleagues selected an RNA aptamer via SELEX that binds with high affinity (K_D_ = 1.65 nM) to the C-terminal region of the N protein of SARS-CoV. By coupling with an antibody to generate an aptamer–antibody hybrid immunoassay, it was possible to detect N protein in concentrations of 2 pg/mL [114]. Soon after, another group reported the selection of an ssDNA aptamer via SELEX that also binds the N protein with a comparable dissociation constant (K_D_ of 4.93 ± 0.30 nM) [115]. Combining nanoparticles with aptamers yielded a quantum dot-conjugated RNA aptamer on a chip, allowing feasible usage for detection of extremely low concentrations (0.1 pg/mL) of SARS-CoV N protein. Upon binding, the aptamer-quantum dot construct emits a fluorescence signal that can be detected via different optical methods [116].

The potential of aptamers for treatment of SARS could also be demonstrated by two groups in 2008. A Korean research team reported the development of a therapeutic RNA aptamer (ES15 RNA) interfering with viral replication by binding the ATPase/Helicase of SARS-CoV. Helicase activity was inhibited up to 85%, with an IC_50_ value of 1.2 nM, subsequently leading to a patent application [117,118]. DNA aptamers to interfere with SARS-CoV helicase activity were selected in the same year. The best hit, aptamer NG8, revealed an IC_50_ value in in vitro assays of 91 nM, and at the same time, a low Michaelis–Menten constant of 5.4 nM, indicating high affinity to its target. Further modification with inverted thymidine increased the inhibitory effect and stability, but slightly decreased affinity [119].

In contrast to SARS-CoV, for MERS-CoV, little is known about aptamer applications. Rutschke and colleagues reported the implementation of a hot-start RT-PCR that included an aptamer, doubling the PCR’s sensitivity [120]. Then again, in the short time window of one to two years, several promising new techniques were developed for COVID-19 diagnostics and therapeutics owing to the greater health burden we are facing during the current pandemic. In the following section, recent aptamer inventions with potential in diagnostics and therapy of SARS-CoV-2 are reviewed.

## 9. Aptamers as Diagnostic Tools for SARS-CoV-2 Monitoring and Detection

Based on the aptamer selected against the SARS-CoV N protein by Cho and collaborators in 2011, a group of Chinese scientists further developed the aptamer and proved the capability to detect SARS-CoV-2 N protein as well. Although cross-reactivity might be an issue and specificity and sensitivity had not been investigated, it proves applicability of aptamers in COVID-19 diagnostics [121].

Besides the N protein, S protein is one of the major targets for diagnosis, treatment, and vaccination due to its importance in virus specificity, host range, and pathogenicity. Song and co-workers identified two aptamers, CoV2-RBD-1C and CoV2-RBD-4C, combining conventional SELEX and computational methods. Both aptamers bound the receptor-binding domain of SARS-CoV-2 S protein with high affinity after optimization (5.8 and 19.9 nM, respectively), which suggests diagnostic and therapeutic potential [122]. A new method termed SENSR was published by Woo and colleagues, who developed a one-pot assay for the fluorescence-based detection of RNA from various pathogens, including SARS-CoV-2. In the presence of target RNA, two probes can hybridize to the RNA and are subsequently ligated by SplintR ligase. T7 RNA polymerase then uses the joined probes as a template to synthesize MG and Broccoli aptamer, capable of stabilizing a fluorogenic dye (i.e., malachite green and DFHBI-1T), which allows fluorescence detection as a readout [123]. In a different study, Wang and colleagues suggested a similar, promising approach involving the CRISPR-Cas13 enzyme and the light-up aptamer “Broccoli” in a two-step recognition process. While in the SENSR method, the probe amplification product is the aptamer itself, here, a substrate is generated that can be bound by a CRISPR RNA. Subsequently, the Cas13 protein is recruited to degrade “Broccoli”, hence destabilizing the fluorophore and essentially reducing the emitted fluorescence. The technique not only allowed detection of virus mutants but also performed well in comparison to RT-qPCR in the detection of virus in human and food samples [124]. Both techniques were applicable for other viruses, including SARS-CoV and MERS-CoV.

Another group reported the selection of four ssDNA aptamers against SARS-CoV-2 N protein, of which the best had a K_D_ value below 0.5 nM. Combined with a hybrid enzyme-linked immunosorbent assay (ELISA), the aptamers formed sandwich-type interactions, resulting in detection of up to 20 pM recombinant protein [125].

Binding of thiolated aptamers (published in [122]) to silver nanoparticles resulted in a device able to detect spike protein binding by measuring the Raman spectrum of the aptasensor. The underlying method, surface-enhanced Raman spectroscopy, allows measurement of the slightest changes in molecule conformation. Thus, detection of virus protein in sub-femtomolar concentration in small sample volumes of 10 µL was possible [111]. The low infrastructural requirements could allow rapid point-of-care testing.

An interesting and unconventional approach utilizes aptamers as biosensors and combines them with already available glucometers applied for diabetes monitoring. Published aptamers with high specificity and affinity towards viral N or S protein were implemented as a molecular switch to initiate the conversion of sucrose to glucose in the presence of viral antigen. In preliminary tests, SARS-CoV-2 infection could be detected in saliva samples [126]. Further development might lead to a ready-to-use product for fast in-house monitoring of infection.

## 10. Aptamers as Therapeutics in COVID-19 Disease

Several potential treatments were suggested for COVID-19 involving aptamers during the past year. At the beginning of this year, two publications demonstrated the power of aptamers selected against the receptor binding domain of the SARS-CoV-2 spike protein as diagnostic and therapeutic tools [51,127]. In the first study, initial screening selected aptamer CoV2-6C3 (Figure 3(3)), which was further modified to improve overall performance. Post-modification, the virus was blocked in the sub-nanomolar range, binding affinity increased to K_D_ = 0.13 nM, and the aptamer was stable in serum and at room temperature for prolonged periods [51]. Soon after, another group published enrichment of potent ssDNA aptamers capable of blocking ligand–receptor interaction, not only preventing host cell infection but also neutralizing the virus in cell culture [127]. Both groups proceeded to in vivo and/or preclinical studies to test the aptamers’ translational potential.

In the work of Pramanik et al., an interesting nano-based ssDNA aptamer system utilizing gold nanostars and the fluorescent dye Rhodamine 6G was developed. The technique not only revealed diagnostic application but also therapeutic potential. On one hand, the system allows detection of a mere 8 virus particles per mL or 130 fg/mL pure antigen. Rhodamine 6G-attached aptamers are conjugated on gold nanostars that quench the dye’s fluorescence. Upon binding to the virus’ spike protein, the aptamer unfolds, distancing the attached fluorophore which nihilates the quenching effect. On the other hand, binding of aptamer-gold nanostar conjugates to the spike protein blocks the ACE2 binding ability and induces membrane damage [128].

Recently, the ssDNA aptamer SP6 was reported to bind SARS-CoV-2 S protein with high affinity and blocked viral infection in cell culture experiments. Interestingly, the binding site of the aptamer is not the receptor-binding domain of the viral spike protein, and further, did not block binding of spike to its ACE2 receptor, thus suggesting a new mode of action. In light of the emerging variants that predominantly contain mutations in the receptor-binding domain of the spike protein, the newly discovered binding site might aid in the development of therapeutical interventions [129].

**Table 1:** Overview of aptamers suggested as diagnostics or therapeutics of coronavirus diseases. N.A.; information not available.

Name	K_D_/K_M_	Type	Length (nt)	Limit of Detection /IC_50_	Target	Reference
SARS-CoV therapeutic aptamer applications						
ES15	N.A.	RNA	107–110	1.2 nM (helicase) 77 nM (ATPase)	SARS-CoV nsp10	[117]
NG8	5.4 nM	DNA	93	91 nM	SARS-CoV helicase	[119]
NG8 modified	26.8 nM	DNA	93	17.5 nM	SARS-CoV helicase	[119]
SARS-CoV diagnostic aptamer applications						
Aptamer 1	1.65 ± 0.41 nM	RNA	83	20 pg/mL (CLISA) 2 pg/mL (nanoarray chip)	SARS-CoV N protein	[114]
Aptamer 1	4.93 ± 0.30 nM	DNA	88	N.A.	SARS-CoV N protein	[115]
Aptamer 1	See [114]	RNA	83	0.1 pg/mL	SARS-CoV N protein	[116]
SARS-CoV-2 therapeutic aptamer applications						
cb-CoV2-6C3	0.13 nM	DNA	46	0.42 ± 0.15 nM (authentic virus)	SARS-CoV-2 S protein	[51]
Aptamer-1	6.05 ± 2.06 nM	DNA	40	5.2 nM (inhibition of binding)	SARS-CoV-2 S protein	[127]
Aptamer-2	6.95 ± 1.10 nM	DNA	40	4.4 nM (inhibition of binding)	SARS-CoV-2 S protein	[127]
Aptamer used from [122]	See [122]	DNA	67	130 fg/mL (antigen) 8 particles/mL (virus)	SARS-CoV-2 S protein	[128]
SP6	21 ± 46 nM	DNA	87	0.2–1 µM (pseudo-virus)	SARS-CoV-2 S protein	[129]
SARS-CoV-2 diagnostic aptamer applications						
Aptamer 1-3	N.A.	DNA	88–57	10 ng/mL (ELAA)	SARS-CoV-2 N protein	[121]
CoV2-RBD-1C	5.8 ± 0.8 nM	DNA	51	N.A.	SARS-CoV-2 S protein	[122]
CoV2-RBD-4C	19.9 ± 2.6 nM	DNA	67	N.A.	SARS-CoV-2 S protein	[122]
MG aptamer	N.A.	RNA	38	0.1 aM (in vitro) 1 aM (clinical samples)	Aptamer stabilizes fluorogenic dye	[123]
Broccoli	N.A.	RNA	65			
Broccoli	N.A.	RNA	65	82 RNA copies (in vitro) 100 RNA copies (SWAB) 500 RNA copies (food)	Aptamer stabilizes fluorogenic dye	[124]
A48	0.49 ± 0.05 nM	DNA	58	20 pM (recombinant protein)	SARS-CoV-2 N protein	[125]
1C,5′(biotin)	See [122] CoV2-RBD-1C	DNA	51	0.03 – 0.32 fM	SARS-CoV-2 S protein	[111]
See [121,122]	N.A.	DNA	94, 57	1.50 pM (N, in vitro) 1.31 pM (S, in vitro) 5.27 pM (N, saliva) 6.31 pM (S, saliva)	SARS-CoV-2 N and S protein	[126]

Since aptamer applications for diagnostics of viral diseases were already reviewed previously [37,38], here, we focus on applications in coronavirus theranostics.

## 11. From Aptamer Selection towards In Vivo Applications Combating Virus Disease

Aptamers have been improved regarding their suitability for in vivo applications. As possible strategies for therapy, aptamers may block the entry of viral particles into host cells by either competing with binding to host cell receptors or virus entry proteins [130]. On the other site, intracellular virus targeting by aptamers is also a promising strategy [131]. Aptamers were developed for blocking nucleocapsid assembly or targeting proteins involved in viral replication, such as SARS CoV NTPase/helicase [117], HIV reverse transcriptase [132], and hepatitis C virus (HCV) RNA polymerase [133], among other examples. To make aptamers prone to therapeutic application, they need to be improved in their thermal stability and resistance against degradation by endogenous nucleases. Exonucleases in the blood have a major contribution to nucleic acid degradation [134]. However, the removal of free ends by circulation of nucleotide sequences enhances their stability, as shown for mRNA [135]. Kim et al. [136] successfully tailored selected RNA sequences against HIV-1 nucleocapsid protein to more stable circular RNA aptamers, thereby maintaining high target binding affinities in the nanomolar range. The approach of aptamer tailoring into circular sequences was further exploited. Kuai and colleagues [137] developed bivalent circular DNA aptamers for in vivo fluorescence imaging of CCRF-CEM tumor-bearing mice, showing the feasibility of their therapeutic use as well as target detection in the living organism. Circular aptamers were also developed as stable probes for targeting Spike RBD of SARS-CoV-2, as already discussed above [51]. Chemical modifications for increasing aptamer stability against nuclease attacks include 2-F, 2′-NH2, or 2′-O-methyl modifications of RNA aptamers (reviewed in [138]). These strategies have their limitation due to the low efficacies of producing 2′-F or 2′-NH2 modified aptamers using in vitro transcription reactions. Additionally, post-SELEX modifications to produce 2′-O-methyl aptamers are needed, which might change aptamer structures. However, another group developed nucleotide polymerase mutants, which tolerate modifications at the 2′-OH ribose group and can be readily used for the selection of nuclease-resistant RNA aptamers [139]. Limitations also account for thioaptamers with phosphorothioate or phosphorodithioate linkages and Spiegelmers, which rely on chemical synthesis procedures [140,141].

Recently introduced aptamer modifications aiming at therapeutic use consist of locked nucleic acids, such as 2′,4′-bridged nucleic acids (BNAs) [142]. Besides their resistance against degradation, these variants have the advantage of entropic stabilization of aptamer structures by methylene bridges. These modifications are not included as a post-SELEX process, which might result in alteration of selected aptamers and loss of binding affinity and selectivity. The development of the X SELEX protocol allows the in vitro selection of BNA aptamers by using genetically engineered polymerases [143]. These enzymes interconvert DNA into LNA and reverse reactions for amplification steps between the SELEX rounds [142]. Aptamers have been improved in their pharmacokinetic properties for increased availability in body fluids, which is necessary for effective virus particle binding or protecting host cell receptors from virus binding. PEGylation, the attachment of poly(ethylene glycol) (PEG), has been the strategy of choice during the last decades. Linear PEG chains, as well as slightly or highly branched PEG moieties, were tested. Oligonucleotides with highly branched PEG moieties are readily taken up by cells, while linear or slightly branched PEG is used for aptamers, which are aimed to bind cells or extracellular targets. Figure 2 depicts some aptamer modifications used for enhancing stability and in vivo utilization of aptamers. Nevertheless, the increase of branching augments oligonucleotide stability in the plasma and prevents rapid aptamer clearance in the kidney and liver [144]. Nanoparticles have been developed for imaging and electrochemical detection, including COVID-19 [145,146]. Aptamer coupled to nanoparticles and selectively recognizing viral particles may be used for in vivo imaging. The nucleolin-recognizing anti-cancer AS1411 aptamer, derivatized with Europium-doped gadolinium oxide (Gd2O3:Eu) nanoparticles [147], as well as the gadolinium-chelate coupled fibrin-binding aptamer for thrombin-clot identification [148], were developed for magnetic resonance imaging (MRI). For the same purpose, superparamagnetic iron oxide nanoparticles (SPION)-deferasirox AS1411 aptamer conjugates were applied for in vivo tumor MRI in a mouse model [149]. Such approaches could be easily extended for in vivo imaging and detection of viral particles. Nanoparticle coupling also influences pharmacokinetic properties and tissue and cell penetration by aptamers, such as lipid nanoparticles (reviewed in [150]). Such lipid-conjugated aptamers have been used for the delivery of siRNAs into target cells aiming at cancer therapy (Fu and Xiang 2020 [151]), but they would also be ideal for interfering with intracellular virus replication or virus capsid assembly. Besides affecting the virus’ life cycle by direct interference with viral proteins, so-called aptazymes produced by aptamer–ribozyme fusions may be delivered into the cells and cleave the genetic material of the virus [152]. Artificial riboswitches for gene expression and replication control of DNA and RNA viruses, which could be delivered by lipid nanoparticles, were developed in the work of Ketzer et al. [153]. The authors showed that aptazyme controlled measles virus fusion expression and diminished infectivity and spreading of the virus.

In summary, while in vivo anti-viral activity of aptamers has not been shown so far, the above-mentioned improvements in aptamer structures and stability will encourage novel therapeutic applications aiming at combating virus infection.

## Data Availability

Not applicable.
